# Update Treatment for HBV Infection and Persistent Risk for Hepatocellular Carcinoma: Prospect for an HBV Cure

**DOI:** 10.3390/diseases6020027

**Published:** 2018-04-20

**Authors:** Joseph Yoo, Hie-Won Hann, Robert Coben, Mitchell Conn, Anthony J. DiMarino

**Affiliations:** 1 Division of Gastroenterology and Hepatology, Department of Medicine, Thomas Jefferson University Hospital, Philadelphia, PA 19107, USA; joseph.yoo@jefferson.edu (J.Y.); robert.coben@jefferson.edu (R.C.); Mitchell.conn@jefferson.edu (M.C.); Anthony.dimarino@jefferson.edu (A.J.D.); 2 Liver Disease Prevention Center Department of Medicine, Thomas Jefferson University Hospital, Philadelphia, PA 19107, USA

**Keywords:** HBV, HCC, anti-HBV therapy, HBV cure risk for HCC

## Abstract

Since the discovery of the hepatitis B virus (HBV) by Blumberg et al. in 1965, its genome, sequence, epidemiology, and hepatocarcinogenesis have been elucidated. Globally, hepatitis B virus (HBV) is still responsible for the majority of hepatocellular carcinoma (HCC). HCC is the sixth-most common cancer in the world and the second-most common cancer death. The ultimate goal of treating HBV infection is the prevention of HCC. Fortunately, anti-HBV treatment with nucleos(t)ide analogues (NAs), which began with lamivudine in 1998, has resulted in remarkable improvements in the survival of patients with chronic hepatitis B and a reduced incidence of HCC. These results were documented with lamivudine, entecavir, and tenofovir. Nonetheless, as the duration of antiviral treatment increases, the risk for HCC still remains despite undetectable HBV DNA in serum, as reported by different investigators with observation up to 4–5 years. In our own experience, we are witnessing the development of HCC in patients who have received antiviral treatment. Some have enjoyed negative serum HBV DNA for over 12 years before developing HCC. Current treatment with NAs can effectively suppress the replication of the virus but cannot eradicate the covalently closed circular DNA (cccDNA) that is within the nucleus of hepatocytes. There still remains a great need for a cure for HBV. Fortunately, several compounds have been identified that have the potential to eradicate HBV, and there are ongoing clinical trials in progress in their early stages.

## 1. Introduction

Since the discovery of the hepatitis B virus (HBV) by Blumberg et al. in 1965 [1], much has been elucidated regarding its genome, sequence, epidemiology, and hepatocarcinogenesis. However, despite all that is known about the virus, and global vaccination programs, HBV infection continues to be a significant public health problem worldwide. It is estimated that there are currently greater than 240–300 million people chronically infected with HBV, and 75% of these individuals live in the Asia-Pacific region. HBV is responsible for 30% of all cases of cirrhosis and over 50% of hepatocellular carcinoma (HCC) with an estimated 50 million new cases diagnosed annually in endemic countries [2,3].

Once infected with HBV, one of the main risks is the development of cirrhosis, hepatic decompensation, and ultimately HCC. Without treatment, 15–40% of these individuals will develop these sequelae during their lifetime [4,5]. Though the exact mechanism is not yet completely understood, the current thought is that the replication of the virus itself is not directly cytotoxic to cells, but rather, it is the response of the host immune system towards infected hepatocytes that ultimately leads to immune mediated liver damage [5,6].

HBV is indeed the most significant hepatocarcinogen responsible for the development of HCC, which currently ranks as the sixth-most common cancer worldwide and the second-most common cause of cancer death [7,8,9,10].

Since the first report by Beasley et al. [4], the connection between HBV and HCC has been well established. Subsequent reports have found that individuals with chronic hepatitis B (CHB) have up to a 30-fold increased risk of HCC, with approximately 25% of individuals infected with CHB developing HCC during their lifetime [5,11,12,13,14,15]. Although most patients with HCC had evidence of cirrhosis [16], HBV has been shown to cause HCC even in the absence of cirrhosis, and inactive carriers of HBV (HBeAg negative, HBV DNA <10,000 copies/mL, normal liver enzymes, no cirrhosis) continue to carry a five-fold increased risk of HCC compared to HBsAg-negative controls [17].

## 2. Antiviral Therapy and Incidence of HCC

Currently-available therapies have shown the ability to delay disease progression and reduce the incidence of HCC with lamivudine, entecavir, and tenofovir disoproxil fumarate [18,19,20]. Furthermore, in patients who have already developed HCC, concomitant antiviral therapy after initial tumor ablation resulted in not only improved survival, but also reduced recurrence of tumor [21,22,23,24,25,26].

However, as the antiviral treatment period is prolonged, the risk for HCC persists in CHB patients even after more than a decade of successful viral suppression as reported earlier [27]. In our experience at the Liver Disease Prevention Center, Division of Gastroenterology and Hepatology of Thomas Jefferson University Hospital, over two thousand patients have been treated for chronic HBV infection over the last twenty-five years. Underlining the risk of HCC, between July 2007 and May 2015, there were 12 patients who developed HCC after having been on anti-viral treatment for over 18 years. The details are shown in Table 1 below.

After the detection of the small tumor, all patients underwent local tumor ablation with continued antiviral therapy and were closely monitored. Five patients developed new or recurrent tumor and eventually died. On the other hand, 7/12 have remained tumor free (Patient 8 underwent liver transplantation) and have continued antiviral therapy.

Our experience is perhaps the longest with oral antiviral therapy since lamivudine, the first nucleoside analogue, was approved in 1998. Given the persistent risk of HCC even after two decades of antiviral (nucleos(t)ides) therapy, there is a desperate need to develop new drugs to cure HBV.

In addition to the persistent risk for an initial diagnosis of HCC, we have also observed the development of de novo or recurrent HCC appearing at 10, 11, and 15 years after the successful ablation of the initial HCC despite continued HBV suppression with antiviral treatment (personal communication).

With the recent development of a cure for the hepatitis C virus, the time now seems ripe to unpack the remaining mysteries of HBV and develop a cure for a virus which continues to cause significant morbidity and mortality worldwide.

## 3. Understanding Hepatitis B Virus

Much of the genome and replication cycle of HBV has been elucidated since its original discovery in 1965 [1], and that understanding has yielded various targeted approaches in the effort to discover a cure. HBV is composed of an outer envelope and a viral nucleocapsid within the envelope. The outer envelope contains three primary envelope glycoproteins, or surface antigens, which are central in the diagnosis of HBV infection [28]. The viral nucleocapsid, or core, contains a DNA polymerase-reverse transcriptase as well as the HBV viral genome, a relaxed partially double-stranded circular DNA molecule (rcDNA) with a length of about 3.2 kb [29,30].

The viral replication cycle begins when HBV recognizes highly-sulfated heparin sulfate proteoglycans (HSPGs) on the surface of liver cells, and binds sodium taurocholate co-transporting polypeptide (NTCP or SLC10A1) to gain entry into the host cell [31,32]. Upon entry into a host cell, the partially double-stranded viral DNA is converted into a covalently closed circular DNA (cccDNA) within the nucleus of the host cell. This stable cccDNA molecule remains within the nucleus of the infected host cell, and is used as the template for the transcription of four viral mRNA intermediates. The mRNA intermediates are then transported to the cytoplasm and translated for the production of seven HBV proteins, including viral surface, core, polymerase, and X proteins. The HBV X protein has been of particular interest as it is felt to play a critical role in the development of HCC, as will be discussed in more detail later. The largest of these mRNA intermediates, the pregenomic RNA (pgRNA) is critical for viral replication as it encodes the viral core, polymerase, and also serves as a template for viral DNA synthesis [5,33,34]. 

## 4. Hepatocarcinogenesis

As previously stated, infection with HBV has a well-established connection with the development of HCC [10,11,12,13,14,15], and HBV DNA sequences have been found to be integrated into the cellular DNA of HCC patients since the early 1980s [35]. Currently, the connection between HBV and HCC has been so firmly established, that HBV is considered a group 1 human carcinogen by the World Health Organization.

Nevertheless, the entire carcinogenic process of HBV-associated HCC is not fully elucidated, and studies have suggested that the mechanism is most likely multifactorial. In addition to the integration of HBV DNA into host chromosomes, there could be the activation of cellular oncogenes, inactivation of tumor suppressor genes, chronic liver injury, inflammation and regeneration (cellular DNA synthesis, impaired cellular repair etc.), activation of cellular proto-oncogenes or suppression of growth regulating genes, and an increase of the HBx protein, which plays an important role in the transcription of HBV DNA as well as in hepatocarcinogenesis. As suggested earlier, the pathogenesis of hepatocellular injury and ultimately cirrhosis seems to result from the host immune response against infected hepatocytes, as opposed to being a result of viral replication itself. Similarly, the carcinogenesis of HBV infection results from creating an environment of chronic inflammation, as well as interactions the virus has with signaling pathways in both the innate and adaptive immune system of the infected host. For example, NF-kB, a signaling molecule involved in the pro-inflammatory response of the innate immune system, and the Tim-3/galectin-9 signaling pathway, associated with the HBV-mediated T cell dysfunction within the adaptive immune system, have both been shown to play a role in the development of HBV-associated HCC [36].

One of the more important factors in the hepatocarcinogenesis of HBV is likely to be the integration of the virus into the host genome. By integrating into the host genome, endogenous genes can be affected and, subsequently, their expression can become dysregulated. One such example is the TERT gene, which encodes telomerase reverse transcriptase, and is often found to be overexpressed in cancer cells [36]. In 2012, Sung et al. [37] published the results of sequencing 81 HBV-positive and seven HBV-negative HCC genomes, along with adjacent normal tissues. The results showed that HBV integration was observed more frequently in the tumors (86.4%) compared to adjacent liver tissues (30.7%). Furthermore, recurrent integration events (≥4) were found at known cancer-related genes, including TERT, resulting in overexpression of these genes in tumor versus normal tissue.

In addition to causing altered expression of endogenous genes by integration into the host genome, the HBV genome itself, and the viral proteins it encodes for, carry properties that increase the risk for HCC. One such property is the relatively high rate of mutation of the HBV genome, and recent work by Park et al. has now identified eight specific mutations within the HBV genome that indicate an increased risk for the development of HCC [36,38]. HBV X (HBx), as previously stated, is a viral protein that is also felt to play an important role in the pathogenesis and hepatocarcinogenesis of HBV. Specifically, it has been shown to interact with Bcl-2, a well-known protein involved in regulating cell death and apoptosis [39]. The connection of HBx protein and HCC is further supported by the fact that the HBx protein is found in most patients with HBV-related HCC, and that the HBx gene is the most frequently integrated viral sequence in HCC [36].

## 5. Current Therapies

Current treatments for hepatitis B include interferon and the nuceleos(t)ide analogs lamivudine, adefovir, entecavir, telbivudine, tenofovir disoproxil fumarate and, recently FDA-approved tenofovir alafenamide. Interferon works primarily through immune modulation and has only a weak antiviral effect, while the nucleos(t)ide analogs suppress the replication of the viral genome by selectively targeting the viral reverse transcriptase [5,40]. Ultimately, the goal of treatment with either interferon or a nucleos(t)ide analog is to achieve a functional cure by preventing the development of cirrhosis and HCC. Indicators of successful therapy (functional cure) are the loss of HBsAg and/or seroconversion to anti-HBs, and undetectable HBV DNA in serum. However, without eradicating cccDNA, HBV continues to carry the risk of development of HCC even if a functional cure with antiviral treatment is achieved, especially in patients who have already developed cirrhosis [27].

## 6. Emerging Therapies

Multiple emerging drug therapies are currently in the early stages of development as part of the growing effort to find a true cure for HBV [41]. Generally speaking, the desired emerging therapies would include: (1) prevention of the entry of the whole virion into an uninfected hepatocyte by blocking the liver specific receptor (sodium taurocholate co-transporting polypeptide (NTCP) [32,42]; (2) prevention of the virion release into circulation; (3) directly targeting cccDNA, such as the formation of, or destabilization and elimination of HBV cccDNA. It is the stable HBV cccDNA, found within the nucleus of infected cells, that stands as the biggest obstacle in finding a cure, as it allows HBV to persist and integrate into the host genome [34]; (4) RNA interference during the transcription of RNA including pregenomic RNA; (5) direct acting antivirals (DAAs) that target the HBV capsid; (6) the conversion of rcDNA to cccDNA; (7) Immune modulation of innate and adaptive immune responses, (such as toll-like receptor agonists, HBV specific T cell vaccine) ; and (8) Inhibition of the viral DNA polymerase (currently done with nucleos(t)ide analogues). Levrero et al. has introduced the recent development of potential cure drugs with detailed updates [43].

## 7. Conclusions

In light of the growing understanding of HBV and the focus on the need for continued HBV basic science research, there still remain several unanswered questions. There are obstacles to solving the remaining questions, such as the current lack of a robust tissue culture system for in vitro studies and the lack of practical animal models for in vivo studies. However, the future is promising, and each remaining unanswered question about HBV represents an unexplored opportunity to discover a new potential strategy in the effort to cure HBV.

## Figures and Tables

**Table 1:** Development of HCC in patients with cirrhosis on long-term antiviral therapy.

Pt	Date startTx	Chang in Child Class On Tx	Date HCC Dx	Yrs on anti-HBV Tx at HCC DX	Yrs with HBV DNA (-)	Age (yr) at HCC Dx	Size (cm) and Site of HCC	HBVDNA at HCC Dx	Anti-HBV Tx	Status
1	4/1998	B→A	7/2007	9.3	3.4*	53	1.1 Junction	UD^#^	LAM + TDF	alive
2	6/2002	A→A	8/2007	5.2	4.7	70	1.0 Rt	UD	LAM + TDF	alive
3	1/1998	B→A	3/2008	10.2	8.2	68	2.8 × 2.5	UD	LAM + TDF	dead
4	5/1998	A→A	2/2008	9.8	6.7	76	1.8 × 0.9 Lt	UD	LAM + TDF	alive
5	7/2004	B→B	9/2009	5.2	4.7	52	3.9 Rt	UD	LAM + TDF	alive
6	7/2001	B→B	9/2010	9.2	4.1	54	2.8 Rt	UD	LAM + TDF	dead
7	2/2004	A→A	6/2013	9. 3	7.7	57	2.5 Lt med	UD	TDF	dead
8	2/1996	A→A	7/2013	17.4	9.7	73	1.6 × 1.4 Rt	UD	TDF	dead
9	8/1997	A→A	6/2014	16.8	5.9	54	2.2 × 1.9 Lt lat	UD	ETV	alive
10	5/1996	A→A	10/2014	18.4	10.4	74	3.4 Rt	UD	LAM + TDF	dead
11	2/2000	A→A	4/2015	15.2	12.4	62	3.4 × 3.4 Rt	UD	TDF	alive
12	2/2000	B→A	5/2015	15.3	12.2	65	3.8 Rt	UD	TDF	alive

LAM, lamivudine, TDF, Tenofovir disoproxil fumarate. UD^#^: undetectable. * pt has been HBV DNA (-) until 3 yrs before HBV DNA became detectable (22 IU/mL) when TDF was added.
